# Bioactive Lipids as Chronic Myeloid Leukemia’s Potential Biomarkers for Disease Progression and Response to Tyrosine Kinase Inhibitors

**DOI:** 10.3389/fimmu.2022.840173

**Published:** 2022-04-13

**Authors:** Felipe Campos de Almeida, Maria G. Berzoti-Coelho, Diana Mota Toro, Maira da Costa Cacemiro, Vitor Leonardo Bassan, Gabriel Dessotti Barretto, Pedro Manoel Marques Garibaldi, Leonardo Carvalho Palma, Lorena Lobo de Figueiredo-Pontes, Carlos Arterio Sorgi, Lucia Helena Faciolli, Luiz Gustavo Gardinassi, Fabíola Attié de Castro

**Affiliations:** ^1^ Department of Clinical Analyses, Toxicology and Food Science, School of Pharmaceutical Sciences of Ribeirão Preto, University of São Paulo, Ribeirão Preto, Brazil; ^2^ Biological Science Institute, Department of Basic and Applied Immunology at Manaus Federal University, Manaus, Brazil; ^3^ Division of Hematology, Department of Medical Imaging, Hematology, and Clinical Oncology, Ribeirão Preto Medical School, University of São Paulo, Ribeirão Preto, Brazil; ^4^ Center for Cell-Based Therapy, Regional Blood Center of Ribeirão Preto Medical School, University of São Paulo, Ribeirão Preto, Brazil; ^5^ Chemistry Department, Faculty of Philosophy, Sciences and Letters at Ribeirão Preto, University of São Paulo, Ribeirão Preto, Brazil; ^6^ Department of Biosciences and Technology, Institute of Tropical Pathology and Public Health, Federal University of Goiás, Goiania, Brazil

**Keywords:** chronic myeloid leukemia, bioactive lipids, tyrosine kinase inhibitors, pathogenesis and metabolomics, tyrosine kinasa inhibitor

## Abstract

Chronic myelogenous leukemia (CML) is a myeloproliferative neoplasm that expresses the Philadelphia chromosome and constitutively activated Bcr-Abl tyrosine kinase in hematopoietic progenitor cells. Bcr-Abl tyrosine-kinase inhibitors (TKI) do not definitively cure all CML patients. The efficacy of TKI is reduced in CML patients in the blastic phase—the most severe phase of the disease—and resistance to this drug has emerged. There is limited knowledge on the underlying mechanisms of disease progression and resistance to TKI beyond *BCR-ABL1*, as well as on the impact of TKI treatment and disease progression on the metabolome of CML patients. The present study reports the metabolomic profiles of CML patients at different phases of the disease treated with TKI. The plasma metabolites from CML patients were analyzed using liquid chromatography, mass spectrometry, and bioinformatics. Distinct metabolic patterns were identified for CML patients at different phases of the disease and for those who were resistant to TKI. The lipid metabolism in CML patients at advanced phases and TKI-resistant patients is reprogrammed, as detected by analysis of metabolomic data. CML patients who were responsive and resistant to TKI therapy exhibited distinct enriched pathways. In addition, ceramide levels were higher and sphingomyelin levels were lower in resistant patients compared with control and CML groups. Taken together, the results here reported established metabolic profiles of CML patients who progressed to advanced phases of the disease and failed to respond to TKI therapy as well as patients in remission. In the future, an expanded study on CML metabolomics may provide new potential prognostic markers for disease progression and response to therapy.

## Introduction

Chronic myelogenous leukemia (CML) is a myeloproliferative neoplasm characterized by the presence of the cytogenetic marker named Philadelphia (Ph) chromosome, which derives from a reciprocal translocation between chromosomes 9 and 22, i.e., t (9; 22) (q34; q11). The presence of Ph results in the production of constitutively activated Bcr-Abl tyrosine kinase in hematopoietic progenitor cells. The disease evolves in three clinical phases of increasing severity: chronic phase (CP), accelerated phase (AP), and blastic phase (BP) (1–3).

The currently available therapies for CML patients in the chronic phase (CML-CP) are chemotherapy with hydroxycarbamide and tyrosine kinase inhibitors (TKI) and allogeneic bone marrow transplantation. TKI induces high rates of cytogenetic and molecular remissions in CML-CP patients at diagnosis (4–6). However, resistance to TKI is a major limitation in the treatment of *BCR-ABL1^+^* leukemias, including CML patients in advanced phases and some CML-CP patients who started the treatment just after diagnosis (4–6).

Several *in vivo* and *in vitro* strategies have been studied to overcome resistance to TKI, such as imatinib dose escalation, a combination of imatinib and chemotherapeutic agents, alternative TKI (nilotinib, dasatinib, ponatinib), immunotherapies, and other Ras signaling pathway inhibitors (4, 5, 7–9). Some clinical features at diagnosis, early response to TKI treatment, the presence of certain kinase domain mutations, and disease clonal evolution may impact the prognosis of CML patients (10). As TKI therapy is not curative, CML patients should use this medication constantly to prevent disease relapse and progression. In this context, many recent studies have demonstrated the possibility and safety of TKI withdrawal in CML patients with a stable deep molecular response to TKI (11).

It remains unclear which cellular and molecular mechanisms underlie disease progression and resistance to TKI, beyond *BCR-ABL1*, as well as how TKI treatment and disease progression affect CML patients’ metabolome. In this sense, the present study aims to describe the global metabolic profile of CML patients in different phases of disease and response to TKI.

Metabolomic approaches contribute to identifying new mechanisms of disease pathogenesis and progression. The present study does not report all metabolites but focuses on bioactive lipids, which are biological molecules involved in cell structure, energy storage, and several biological processes including signaling, protein trafficking, cell death, and cell proliferation (12, 13).

Changes in lipid metabolism in neoplasia, called “lipid metabolic reprogramming”, influence cell cycle, proliferation, growth, and differentiation and lead to carcinogenesis (13). Identification of lipid metabolism modifications in hematological cancer helps to identify novel biomarkers and metabolic targets for therapy. The CML metabolomic analysis may promote early detection of disease progression, TKI response, or disease relapse in patients, considering the lipids or molecules that represent key signaling pathways or biomarkers involved in disease processes.

## Subjects and Methods

### Subjects

This study enrolled 9 healthy subjects and 29 CML patients (10 in the chronic phase, 13 in the advanced phase—accelerated and blastic phases—and 6 with complete molecular remission post-TKI therapy) who signed an informed consent form before blood collection. The control group included 6 men and 4 women with a mean age of 46.4 years (range, 23–68 years), while the CML group was composed of 17 men and 12 women, with a mean age of 49.79 years (range, 20–76 years).

The CML patients were on regular follow-up from 2015 to 2019 at the Division of Hematology, University Hospital of the Ribeirão Preto Medical School (HC-FMRP-USP), State of São Paulo, Brazil, and their clinical features are reported in **Table 1**. The CML diagnosis was confirmed by the detection of the Ph chromosome in the bone marrow cytogenetic analysis and/or *BCR-ABL1* rearrangement. This study was approved by the Ethics Committee for Human Research from the School of Pharmaceutical Sciences of Ribeirão Preto and HC-FMRP-USP, protocol No. 398/2016. Informed consent was obtained from all participants from this study.

**Table 1:** Clinical and socio-demographic features of CML patients and controls.

Sample	Gender	Race	Age	Diagnosis	Treatment	Response
1	F	Yellow	63	CML Chronic Phase	At diagnosis	
2	M	White	61	CML Chronic Phase	At diagnosis	
3	M	White	57	CML Chronic Phase	At diagnosis	
4	F	White	31	CML Chronic Phase	At diagnosis	
5	M	Black	40	CML Chronic Phase	At diagnosis	
6	F	White	68	CML Chronic Phase	At diagnosis	
7	F	White	74	CML Chronic Phase	At diagnosis	
8	M	White	52	CML Chronic Phase	At diagnosis	
9	M	White	38	CML Chronic Phase	At diagnosis	
10	M	White	20	CML Chronic Phase	At diagnosis	
11	M	White	29	CML Chronic Phase	Imatinib	CCyR/CMR
12	M	White	56	CML Chronic Phase	Dasatinib	CCyR/CMR
13	M	White	45	CML Chronic Phase	Dasatinib	CCyR/CMR
14	M	White	56	CML Chronic Phase	Imatinib	CCyR/CMR
15	F	White	47	CML Chronic Phase	Imatinib	MR 5.0
16	F	White	60	CML Chronic Phase	Dasatinib	MR 5.1
17	F	White	41	CML Advanced Phase	Dasatinib	Resistant
18	M	White	32	CML Advanced Phase	Dasatinib	Resistant
19	M	White	53	CML Advanced Phase	Nilotinib	Resistant
20	F	White	69	CML Advanced Phase	Dasatinib	Resistant
21	M	White	34	CML Advanced Phase	Dasatinib	Resistant
22	F	White	64	CML Advanced Phase	Nilotinib	Resistant
23	M	Black	28	CML Advanced Phase	Dasatinib	CCyR/CMR
24	M	White	33	CML Advanced Phase	Dasatinib	CMR
25	M	White	63	CML Advanced Phase	Dasatinib	CCyR/CMR
26	F	White	62	CML Advanced Phase	Imatinib	Resistant
27	F	Black	63	CML Advanced Phase	Nilotinib	Resistant
28	F	White	76	CML Advanced Phase	Dasatinib	Resistant
29	M	Black	29	CML Advanced Phase	At diagnosis	
30	F	White	41	Control		
31	M	White	54	Control		
32	M	White	23	Control		
33	F	Black	47	Control		
34	M	White	42	Control		
35	M	White	51	Control		
36	M	White	68	Control		
37	M	White	50	Control		
38	F	Black	54	Control		
39	F	White	34	Control		

F, female; M, male; CCyR, Complete cytogenetic response; CMR, complete molecular response; MR, molecular response.

### Plasma Isolation

Twenty milliliters of peripheral blood was collected from CML patients and healthy subjects, and plasma was separated by centrifugation at 400×*g* for 10 min at 4°C. Aliquots were stored at −80°C for further analysis of bioactive lipids.

### Quantification of Sphingolipids by LC-MS/MS

Briefly, the HPLC Nexera X2 (Shimadzu, Kyoto, HO, JP) coupled to the AB Sciex QTrap triple-quadrupole mass spectrometer were used for positive ESI LC-MS/MS analysis. HPLC analysis was performed using an Ascentis Express C18 column, at 35°C, previously equilibrated for 5 min with 30% methanol and 70% formic acid (1% in ddH_2_O, v/v). One minute after starting the HPLC program, 10 μl of the sample was injected and analyzed under a flow rate of 0.3 ml/min, using the following setup: 0.0–1 min (30% methanol), 1.1–2.5 min (85% methanol), 2.5–5.0 min (100% methanol), 5.0–15 min (hold 100% methanol), and 15.1–20 min (re-equilibrate with 30% methanol and 70% formic acid). The ion source conditions and gas parameters were set as follows: ion spray voltage = 4,500 V, ion source heater temperature = 450°C, collision gas = medium, ion source gas 1 = 30 psi, ion source gas 2 = 60 psi, and curtain gas = 45 psi.

### Lipid Quantification by LC-MS/MS

Plasma samples were used for lipid extraction and quantification and processed as described by Sorgi et al. (2018) (14). Briefly, 250 μl of each plasma sample was mixed with 1 ml of methanol and 10 μl of internal standard and centrifuged at 500×*g*, at 4°C, for 10 min to remove the precipitate. The supernatant was transferred to a 15-ml tube and mixed with 8.75 ml of DEPC water. Meanwhile, a syringe was coupled to the C18 column and slotted in the vacuum system adjusted to 10 kPa. Then, 2 ml of methanol was injected into the column, followed by 2 ml of 0.1% acetic acid in DEPC water. The samples were injected into the column, followed by a wash with 0.1% acetic acid in DEPC water, and eluted with 1 ml of 0.1% acetic acid in methanol in previously labeled 1.5-ml vials. Subsequently, the samples were dehydrated in the vacuum dryer at room temperature and dissolved in methanol/DEPC water (70/30, v/v). Finally, 50 μl of the samples was transferred to autosampler vials for LC-MS analysis. The HPLC Nexera X2 (Shimadzu, Kyoto, HO, JP) coupled with the AB Sciex QTrap triple-quadrupole mass spectrometer was used for positive ESI LC-MS/MS analysis. HPLC analysis was carried out using an Ascentis Express C18 column, at 35°C. Mass spectral data were acquired with negative electrospray ionization, and the full scan of mass-to-charge ratio (m/z) ranged from 100 to 1500. The Protein Wizard software was used to convert.wiff into mzXML files (15).

### Bioinformatic Analysis

The R package apLCMS software was used for peak peaking, noise filtering, alignment of retention time and mass-to-charge ratio (*m/z*), and quantification of metabolite features, which are defined as *m/z*, retention time, and intensity (16). Data were log2 transformed and normalized to the mean. Features detected in 50% of the samples were used for statistical analysis. The mummichog software (version 2) was used for metabolic pathway enrichment analysis (mass accuracy under 10 ppm), whereas tentative metabolite annotations include multiple ions and adducts. Heatmaps were plotted with the R package gplots. Hierarchical clustering was performed with the R package amap with Spearman distance method and ward linkage algorithm. Volcano and bubble plots were plotted with the aid of the package ggplot2.

### Statistical Analysis

To analyze metabolomics data, significant differences were detected with the R package limma, while false discovery rate was calculated using the Benjamini–Hochberg method.

## Results and Discussion

Bcr-Abl oncoprotein promotes malignant transformation by altering cellular adhesion properties, stimulating mitogenic signaling pathways, and inhibiting programmed cell death (17–19). Bcr-Abl TKI approval was a great success in disease therapy by reducing the mortality rate associated with CML, and it revolutionized treatment (6, 20). It induces a high rate of cytogenetic and deep molecular response in CML-CP patients (4–6, 17). Despite their outstanding activity against CML, TKI does not cure the disease, and the patients require indefinite treatment. Several studies have examined the possibility of TKI therapy discontinuation in CML patients without relapse to keep them in CML remission for long periods. This treatment-free remission has several benefits for CML patients, such as improved quality of life, lower treatment costs, and fewer side effects and complications (21).

The knowledge on the cellular and molecular mechanisms involved in disease progression and resistance to TKI, beyond *BCR-ABL1*, is limited. The impact of TKI treatment and disease progression on the metabolome of CML patients is not fully elucidated. Here, we describe the metabolomic profiles, especially of bioactive lipids, in CML patients in different phases of disease and treated with TKI. In this scenario, the use of a novel tool, such as metabolomics, may be useful to identify new metabolic pathways related to disease progression and response to TKI. Metabolites are the output of cellular and molecular metabolism that account for the expression and activity of genes, transcripts, and proteins and may help to comprehend the processes linked to CML pathogenesis and progression and the patient’s response to TKI.

**Figure 1:**
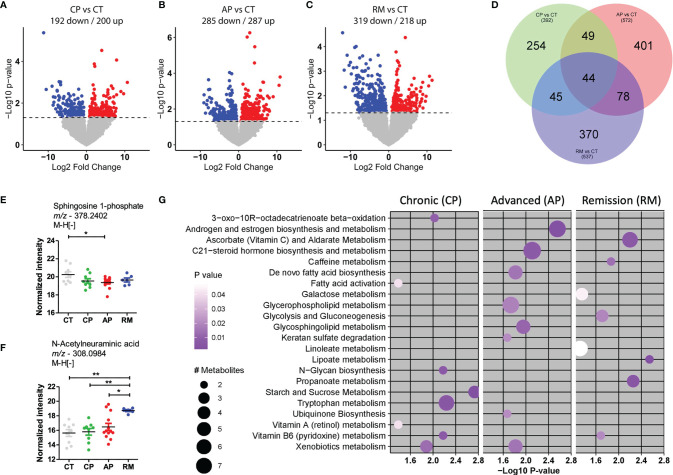
Metabolites differentially abundant in CML patients in comparison with the control group. **(A–C)** Volcano plots to compare down- and upregulated metabolites in **(A)**. CML patients in the chronic phase (CP; *n* = 10) and control group (CT; *n* = 9); **(B)** CML patients in the advanced phase (AP, *n* = 13) and control group (CT); **(C)** CML patients in remission post-TKI therapy (RM; *n* = 6) and control group (CT). **(D)** Venn diagram of differentially abundant metabolites in each group, after comparison. **(E, F)** Scatter-plots of the amount of top predicted metabolites in CML patients and the control group. Sphingosine-1-phosphate was less abundant in CML-AP patients than in the control group. N-Acetylneuraminic acid was more abundant in patients in remission than in control subjects and CML-CP and CML-AP patients. **(G)** The most representative metabolic pathways in CML patients, compared with the control group. The circle size represents the number of differentially abundant metabolites, and the circle color means the degree of significance (*p*-value magnitude) after comparison among groups. Significant metabolite features were identified by ANOVA with repeated measures, associated with Tukey’s multiple comparisons test (^*^ *p* < 0.05; ^**^ *p* < 0.01).

In the present study, we used a high-resolution metabolomics platform in which we measured 8,340 metabolite features. After processing data and performing quality control, we selected 8,174 features for subsequent analysis. The control group was compared with CML-CP and CML-AP patients, patients resistant to TKI, and patients in remission post-TKI therapy. The differentiated metabolites among these groups were extracted by univariate statistical criteria (*p*-value <0.05) and depicted in the volcano plot (**Figures 1A–C**). Compared with the control group, CMP-AP patients had more upregulated genes than the other groups, while patients in remission had more downregulated genes than the other groups. Forty-four predicted metabolites were consistently modulated for each group, compared with the controls using the Venn diagram (**Figure 1D**). We compared the amount of these metabolites among the studied groups in order to identify their biological relevance (**Figures 1E, F**).

The predicted sphingosine 1-phosphate (S1P) was less abundant in CML-AP patients than in the control group. Sphingosine kinase-1 (SphK1)—an enzyme that phosphorylates sphingosine into S1P—overexpression in CML cell lines resistant to TKI protects them against imatinib therapy by blocking the mitochondrial release of caspase activators (22). In acute myeloid leukemia and lymphoma, S1P stimulates the growth and survival of neoplastic cells (23). Such reports contrast to our findings that S1P was decreased in CML-AP patients. However, the literature reports performed a lipidomic analysis in cell lines while the present study used plasma samples that have a richer lipid environment and interferences from other cells’ lipid content.

N-Acetylneuramic acid (sialic acid) was another metabolite differentially detected in CML patients. Sialic acids are highly expressed in solid and hematological tumor cells. The high expression of sialic acids is associated with tumorigenesis and tumor progression by contributing to tumor cell escape from apoptosis, immune response, and resistance to chemotherapy (24). In our study, sialic acid was predicted as more abundant in CML patients than in the control group. Interestingly, the sialic acid concentration was increased in CML patients in remission post-TKI, suggesting that they were released from the membrane of leukemic cells after TKI treatment.

To examine which metabolic pathways underlay these significant metabolite features, we used *mummichog*, a software specifically designed for untargeted metabolomics. In CML-AP patients, significant features were enriched for pathways involved in androgen and estrogen biosynthesis and metabolism, C21-steroid hormone biosynthesis and metabolism, *de novo* fatty acid biosynthesis, glycerophospholipid metabolism, glycosphingolipid metabolism, keratan sulfate degradation, ubiquinone biosynthesis, and xenobiotics metabolism (**Figure 1G**). The metabolic pathways of ascorbate (vitamin C) and aldarate, caffeine, galactose, glycolysis and gluconeogenesis, linoleate, lipoate, and propanoate were associated with CML patients in remission post-TKI treatment (**Figure 1G**). Vitamin B6 (pyridoxine) metabolism was associated with CML patients in chronic phase and remission post-TKI therapy (**Figure 1G**). Compared with the control group, CML-CP and CML-AP patients had enriched significant metabolite features in xenobiotic metabolism (**Figure 1G**). The xenobiotic-metabolizing enzymes mediate the elimination of drugs from inside the cell. Upregulation of the efflux transporter ABCC3 correlates with a poor prognosis for CML-CP patients (25). The altered expression of an efflux transporter in CML-AP patients (26) contributes to resistance to TKI and disease progression. Corroborating these reports, we detected enrichment of efflux transporter metabolites mainly in TKI-resistant patients and advanced phases of CML. Such enrichment might be one of the mechanisms by which these patients were unlikely to acquire molecular response and progress to the blastic phase.

**Figure 2:**
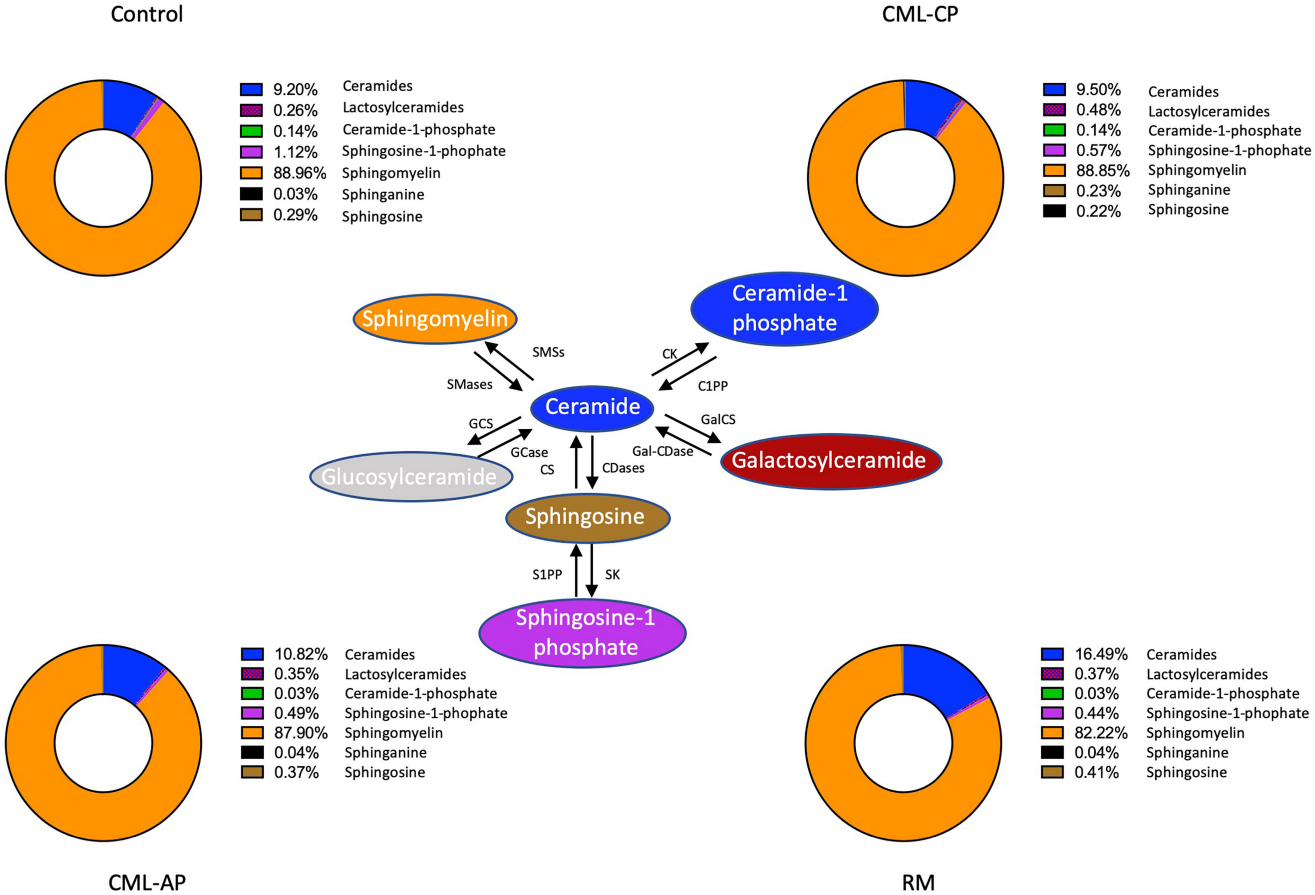
Sphingolipid profile in chronic myeloid leukemia patients. Relative frequency of sphingolipid classes in the control group and patients in the chronic (CP) and advanced (AP) phases of the disease and in remission post-TKI therapy (RM). SMSs, Sphingomyelin synthase; SMases, Sphingomyelinase; CK, ceramide kinase; C1PP, ceramide-1-phosphate phosphatase; GCS, glucosylceramide synthase; GCases, Glucocerebrosidase; CS, ceramide synthases; CDases, ceramidases; GalCS, galactosylceramide synthase; Gal-CDase, galactosylceramidase; SK, sphingosine kinase; S1PP, spingosine-1-phosphate phosphatase.

We used mass spectrometry to analyze the sphingolipid profile of the control group and CML patients at different phases of the disease. The relative frequency of sphingolipid subclasses is depicted in **Figure 2**. Interestingly, the frequency of ceramides in remission patients (16.49%) differed from that of the control group (9.20%) and patients in chronic (9.50%) and advanced phases (10.82%). CML patients responsive to TKI therapy had a lower frequency of sphingomyelin (82.22%) when compared with the control group (88.96%) and patients in chronic (88.85%) and advanced (87.90%) phases. Therefore, it seemed that remission patients had a distinct sphingolipid profile, with an accumulation of ceramides and impaired conversion into sphingomyelin (**Figure 2**).

It is well-known that ceramides mediate induction of apoptosis and inhibition of cell proliferation (27). TKI therapy upregulates ceramide synthases (CerS) genes and downregulates antiapoptotic SphK1 in cell lines, while targeting sphingolipids towards the accumulation of ceramides improves TKI therapy (28). CML cell lines treated with ceramide analogs remain sensitive to apoptosis (27). Treatment with imatinib enhances the abundance of ceramides in sensitive cells but not in resistant cells (29), corroborating our findings. Bcr-Abl is able to target sphingomyelin synthase 1 (Sms1), an enzyme that converts ceramide into sphingomyelin, enhancing its conversion, and favors cell proliferation (30). Thus, these reports corroborate our findings that patients in remission post-TKI therapy had a lower abundance of sphingomyelin.

**Figure 3:**
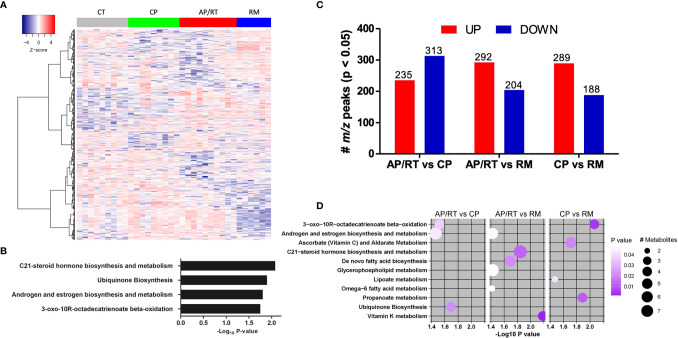
Dynamic of the abundance of metabolite features associated with disease progression and response to TKI therapy. **(A)** One-way hierarchical clustering based on the intensity of highly significant metabolite features selected by ANOVA. **(B)** Metabolic pathways enriched by significant metabolite features. **(C)** Up- and downregulated abundant metabolites in patients in advanced (AP)/resistant (RT) vs. chronic phase (CP), AP/RT vs. remission post-TKI (RM), and CP vs. RM. **(D)** Metabolic pathways enriched by significant metabolite features. The circle size represents the number of differentially abundant metabolites, and the circle color means the degree of significance (*p*-value magnitude) after comparison between the control and CML patient groups.

Based on the distinct metabolite components, a heatmap of hierarchical clustering analysis was created using ANOVA analysis (**Figure 3A**) that included only CML-AP patients who were resistant to TKI therapy. A well discriminatory cluster was found between the CML groups and control in the heatmap, mainly in the remission group. The *mummichog* enrichment algorithm was also used to analyze enrichment pathways. Interestingly, the enriched pathways were C21-steroid hormone biosynthesis and metabolism, ubiquinone biosynthesis, androgen and estrogen biosynthesis and metabolism, and 3-*oxo*-10R-octadecatrienoate beta-oxidation (**Figure 3B**).

We used the same approach reported above to determine the differential abundance of metabolites among CML patients at different phases of the disease and patients resistant to TKI. Compared with CML-CP patients, CML-AP patients who were TKI-resistant had 235 m/z upregulated and 313 m/z downregulated peaks (**Figure 3C**). We used the *mummichog* enrichment algorithm and identified significant features enriched for pathways involved in 3-oxo-10R-octadecatrienoate beta-oxidation, androgen and estrogen biosynthesis and metabolism, and ubiquinone biosynthesis (**Figure 3D**). When CML-AP patients resistant to TKI were compared with CML patients in remission post-TKI therapy, 292 and 204 m/z peaks were up- and downregulated, respectively (**Figure 3C**). Specifically, the enriched metabolite pathways between these two groups were androgen and estrogen biosynthesis and metabolism, C21-steroid hormone biosynthesis and metabolism, *de novo* fatty acid biosynthesis, glycerophospholipid metabolism, omega-6 fatty acid metabolism, and vitamin K metabolism (**Figure 3D**). Compared with CML-CP patients, CML patients in remission had 289 and 188 m/z peaks up- and downregulated, respectively (**Figure 3C**). The enriched pathways were 3-oxo-10R-octadecatrienoate beta-oxidation, ascorbate (vitamin C) and aldarate metabolism, lipoate metabolism, and propanoate metabolism (**Figure 3D**).

It is well-known that sphingolipid metabolism is impaired in hematological malignancies and can lead to drug resistance. Ceramide is the main component of sphingolipid metabolism and displays proapoptotic effects (31). In fact, ceramide and sphingosine inhibit proliferation and have proapoptotic effects, but S1P induces cell growth and exerts antiapoptotic action (32). Ceramide analogs were able to decrease cell proliferation and induce apoptosis in CML cell lines. Furthermore, in the same study, ceramide accumulation enhances proapoptotic effects of the second-generation TKI dasatinib (20). Also, the SPHK1 inhibitor named SKI-II acts synergistically with imatinib to inhibit cell growth and survival in primary CML cell lines (21). Therefore, upregulation of ceramide levels and downregulation of antiapoptotic lipid mediators might generate a more efficient approach to eliminate CML cells either in combination with TKI or by itself.

In summary, we defined the metabolomics profile from CML patients’ plasma and associated the results with different phases of the disease and response to TKI treatment. Our data supported that detection and monitoring of differential abundant metabolites in CML patients shall be a useful tool to describe new biomarkers and attract candidates for devising new targeted and combined therapies in CML. To date, Bcr-Abl is the only biomarker for the diagnosis and prognosis of CML. In this context, the lipid profile may be a criterion to select patients who are unlikely to respond to TKI therapy and could benefit from therapy using imatinib in combination with new drugs or different TKI.

## Data Availability Statement

The raw data supporting the conclusions of this article will be made available by the authors, without undue reservation.

## Ethics Statement

The studies involving human participants were reviewed and approved by the Ethics Committee for Human Research from the School of Pharmaceutical Sciences of Ribeirão Preto and HC-FMRP-USP, protocol No. 398/2016. The patients/participants provided their written informed consent to participate in this study.

## Author Contributions

FCA designed and performed experiments, analyzed data and wrote the paper. MGBC, MCC, VLB, GDB performed experiments of the cell and plasma isolation. LGG performed all the bioinformatics analysis. FCA, DMT and CAS performed all lipid quantification by LC-MS/MS. PG, LP and LLFP selected and diagnosed the MPN patients included in this study. FCA, LLFP, CAS, LHF, LGG and FAC conceived the project, designed the study, discussed the results, searched for funding and wrote the paper. All authors critically reviewed the manuscript.

## Funding

This study was supported in part by the Coordination for the Improvement of Higher Education Personnel (CAPES; Finance Code 001), the São Paulo Research Foundation (FAPESP; Research grant #2018/19714-7; CTC grant #2013/08135-2; INCTC grant #2014/50947-7; Young Investigator grant #2015/21866-1), and the National Council for Scientific and Technological Development (CNPq; grants #163064/2018-0, #169093/2018-2, #305959/2018-2, and #303259/2020-5). MCC, MGBC and LHF were recipients from FAPESP scholarships (grants #2018/01756-5, #2015/23555-3 and EMU #2015/00658-1, respectively).

## Conflict of Interest

The authors declare that the research was conducted in the absence of any commercial or financial relationships that could be construed as a potential conflict of interest.

## Publisher’s Note

All claims expressed in this article are solely those of the authors and do not necessarily represent those of their affiliated organizations, or those of the publisher, the editors and the reviewers. Any product that may be evaluated in this article, or claim that may be made by its manufacturer, is not guaranteed or endorsed by the publisher.
